# Evaluation of the surface characteristics and antibacterial properties of Titanium dioxide nanotube and methacryloyloxyethylphosphorylcholine (MPC) coated orthodontic brackets-a comparative invitro study

**DOI:** 10.1007/s00784-024-05655-w

**Published:** 2024-05-18

**Authors:** Madhura Rao, M V Ashith, Ethel Suman, Arun M Isloor, Neetha J Shetty, Srikant Natarajan

**Affiliations:** 1 Department of Orthodontics and Dentofacial Orthopaedics, Manipal College of Dental Sciences Mangalore, Manipal Academy of Higher Education, Karnataka, Manipal, 576104 India; 2 Department Of Orthodontics and Dentofacial Orthopaedics, Manipal College of Dental Sciences Mangalore, Manipal Academy of Higher Education, Karnataka Manipal, 576104 India; 3 Department of Microbiology, Kasturba Medical College Mangalore, Manipal Academy of Higher Education, Karnataka Manipal, 576104 India; 4 Department Of Chemistry, National Institute of Technology, Surathkal, 575025 Karnataka India; 5 Department Of Periodontics Manipal College of Dental Sciences Mangalore, Manipal Academy of Higher Education, Karnataka, Manipal, 576104 India; 6 Department Of Oral Pathology Manipal College of Dental Sciences Mangalore, Manipal Academy of Higher Education, Karnataka, Manipal, 576104 India

**Keywords:** Methacryloyloxyethylphosphorylcholine, Stainless steel orthodontic brackets, Streptococcus mutans, Titanium dioxide nanotube, White spot lesions

## Abstract

**Objectives:**

White spot lesions are the most common iatrogenic effect observed during orthodontic treatment. This study aimed to compare the surface characteristics and antibacterial action of uncoated and coated orthodontic brackets.

**Materials and methods:**

Sixty commercially available stainless steel brackets were coated with TiO_2_ nanotubes and methacryloyloxyethylphosphorylcholine. The sample was divided into Group 1: uncoated orthodontic brackets, Group 2: Stainless steel brackets with TiO_2_ nanotubes coating, Group 3: Stainless steel brackets with methacryloyloxyethylphosphorylcholine coating, and Group 4: Stainless steel brackets with TiO_2_ nanotubes combined with methacryloyloxyethylphosphorylcholine coating. Surface characterization was assessed using atomic force microscopy and scanning electron microscopy. *Streptococcus mutans* was selected to test the antibacterial ability of the orthodontic brackets, total bacterial adhesion and bacterial viability were assessed. The brackets were subjected to scanning electron microscopy to detect the presence of biofilm.

**Results:**

The surface roughness was the greatest in Group 1 and least in Group 2 followed by Group 4 and Group 3 coated brackets. The optical density values were highest in Group 1 and lowest in Group 4. Comparison of colony counts revealed high counts in Group 1 and low counts in Group 4. A positive correlation between surface roughness and colony counts was obtained, however, was not statistically significant.

**Conclusions:**

The coated orthodontic brackets exhibited less surface roughness than the uncoated orthodontic brackets. Group 4 coated orthodontic brackets showed the best antibacterial properties.

**Clinical relevance:**

Coated orthodontic brackets prevent adhesion of *streptococcus mutans* and reduces plaque accumulation around the brackets thereby preventing formation of white spot lesions during orthodontic treatment.

## Introduction

Dentistry is a profession where in the primary goal is to provide patients with high-quality oral health care. The field of orthodontics frequently uses brackets and wires as part of fixed orthodontic appliance therapy to correct misaligned teeth, jaws, and bite patterns in most patients. Irregularly arranged upper and lower teeth can have a significant influence on individuals’ oral and dental health, as well as aesthetic appearance [1]. 

The prevalence of these white spot lesions varies from 4.9 to 84% of tooth surfaces, according to studies conducted by Gorelick L et al. and Mizrahi E [2, 3]. The placement of orthodontic archwire brackets, molar bands, elastic/steel ligatures, and other orthodontic appliances make tooth cleaning difficult, creating stagnation areas for plaque and encouraging the colonization of aciduric bacteria, eventually resulting in active white spot lesions that, if left untreated, can progress to a carious lesion [4, 5]. 

*Streptococcus mutans*, which is the regular resident of the oral cavity, plays an important role in the initiation and progression of white spot lesions. *Streptococcus mutans* is a gram-positive bacterium that thrives at temperatures ranging from 18 to 40 degrees Celsius in the oral cavity. This temperature range can cause bacteria to metabolize different kinds of carbohydrates, thereby generating an acidic environment in the oral cavity and eventually leading to dental caries. Of all oral *Streptococci, Streptococcus mutans* is proven to be the most cariogenic. To overcome the inevitable poor oral hygiene occurring due to the use of orthodontic appliances, different surface treatments and coatings have been developed to refine the surface properties, rendering these materials more satisfactory for orthodontic implementation [6]. In the past, both thermal and chemical methods for surface treatments have been used for antibacterial coating of the orthodontic materials. Few of the nanoparticles that have been incorporated into the orthodontic materials include silver, zinc, titanium, copper, magnesium, chitosan and zwitter ion based nanoparticles [7]. Atomic layer deposition, radical graft polymerisation have been used to coat the MPC on the stainless [8, 9]. . 4-Methacryloyloxyethyl trimellitate anhydride (4-META)/methyl methacrylate (MMA) polymer was also used as a binder for the MPC to improve the surface characteristics of the stainless steel [10]. In the past, methods to fabricate TiO_2_ NT by electrodeposition, hydrothermal deposition, anodization and physical vapor deposition have been tried [11, 12]. Some of the methods by which polydopamine have been coated on to the metal surface include a one-step electrodeposition, thermal treatment and oxidant induced polymerisation [13, 14]. 

Polydopamine was used as a binder for binding the coatings to the orthodontic brackets. The catechol moieties are responsible for the ability of polydopamine (PDA) to attach to various types of surfaces, including those beneath water. This property makes PDA a very interesting polymer. The polymer produced by dopamine oxidation includes pyrroles to a lesser extent in addition to indole and dopamine units in several oxidation states. The complicated oxidation-related chemical properties of polydopamine are due to the oxidized o-quinone and o-hydroquinone groups [15, 16]. Furthermore, it has been illustrated that reactive oxygen species are produced by the oxidation of dopamine. These species are bactericidal to both gram-negative and gram-positive bacteria [15]. As a result, the development and survival of microorganisms are impacted by the coating of such compounds on surfaces. Su et al. have expanded the application of polydopamine by creating a convenient shaking-assisted method that creates roughened polydopamine (rPDA) coatings on a range of substrates [17]. The anticipated rPDA coatings demonstrated noticeably improved antibacterial efficacy against both gram-negative and gram-positive bacteria in the absence of an external antibacterial agent [18]. In this study, the same shaking-assisted method was used to coat the orthodontic brackets with PDA.

Medical devices coated with methacryloyloxyethylphosphorylcholine (MPC) demonstrate antibacterial effects. The polymer MPC contains a zwitterionic phosphorylcholine headgroup, preventing nonspecific protein adsorption (protein repellent). MPC polymers generate biocompatible interfaces and surfaces with high antibiofouling performance for both microscopic and macroscopic applications [19]. The hydrophilic polymer 2-methacryloyloxyethyl phosphorylcholine (MPC) with free water surrounding the phosphorylcholine group helps in the detachment and repellence of proteins, thus limiting microbial adhesion [20, 21]. 

TiO_2_NT arrays are three-dimensional nanomaterials. At low manufacturing costs, they have excellent chemical and physical properties. The antibacterial mechanisms of TiO_2_NT are complex and comprise surface roughness variation, charge repulsion and membrane stretching. Surface roughness is responsible for the antibacterial property of TiO_2_NT. First, it can impact surface wettability. The hydrophobic surfaces adhere to other hydrophobic surfaces as the hydrophilic structure adsorbs water film. It is then eventually eliminated. The improved surface hydrophilicity and prevention of hydrophobic bacteria from adhering to TiO_2_NT is attributed to the increased surface roughness of TiO_2_NT. Second, the effects of surface roughness on antibacterial characteristics at the nanoscale or microscale are entirely different [22]. As the surface roughness increases in the nanoscale (10–100 nm) dimensions, bacterial adhesion decreases. However, at the micron level, the bacterial adhesion improves in parallel with the surface roughness. Attachment point theory can explain this phenomenon. According to this theory, increasing roughness creates additional attachment points as well as microscale refuge shelters for bacteria smaller than the surface microtexture, which protect these bacteria from hydrodynamic shear forces [23, 24]. 

To address to this iatrogenic effect of white spot lesions during orthodontic treatment, we decided to use TiO_2_ nanotubes and methacryloyloxyethylphosphorylcholine to coat the brackets with the help of polydopamine as the binder. The TiO_2_ nanotubes are known to have a high specific surface area, high chemical stability and catalytic activity, strong metal support interaction, and excellent performance in alkali and acidic environments making it suitable for the oral environment [12]. Methacryloyloxyethylphosphorylcholine is a zwitterion polymer with protein repellent properties and prevention of biofilm formation while maintaining biocompatibility and has been widely studied with its incorporation into various dental materials [8]. The coatings were chosen due to their superior biocompatibility and increased stability in the oral environment when compared to other nanoparticles [8, 12, 14]. The aim of the study is to evaluate the surface characteristics and antibacterial properties of uncoated and coated orthodontic brackets. Our null hypothesis is that the coated orthodontic brackets do not have reduced surface roughness or improved antibacterial properties.

## Materials and methods

### Sample size calculation

The sample size was calculated using the formula.

**Fig. 1:**
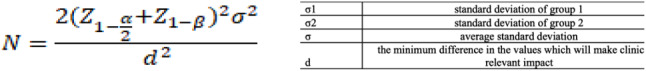
Formula

Based on the Table 4 from the article titled “In vitro assessment of photocatalytic titanium oxide surface modified stainless steel orthodontic brackets for anti-adherent and antibacterial properties against Lactobacillus acidophilus,” [25] comparing the control group and sample group with the key parameter of assessing antibacterial action, with a 1% alpha error, 95% power of the study and clinically significant difference of 20 units, the required sample was 60 with 4 groups consisting of 15 samples each (Fig. 1).

### Sample preparation

**Fig. 2:**
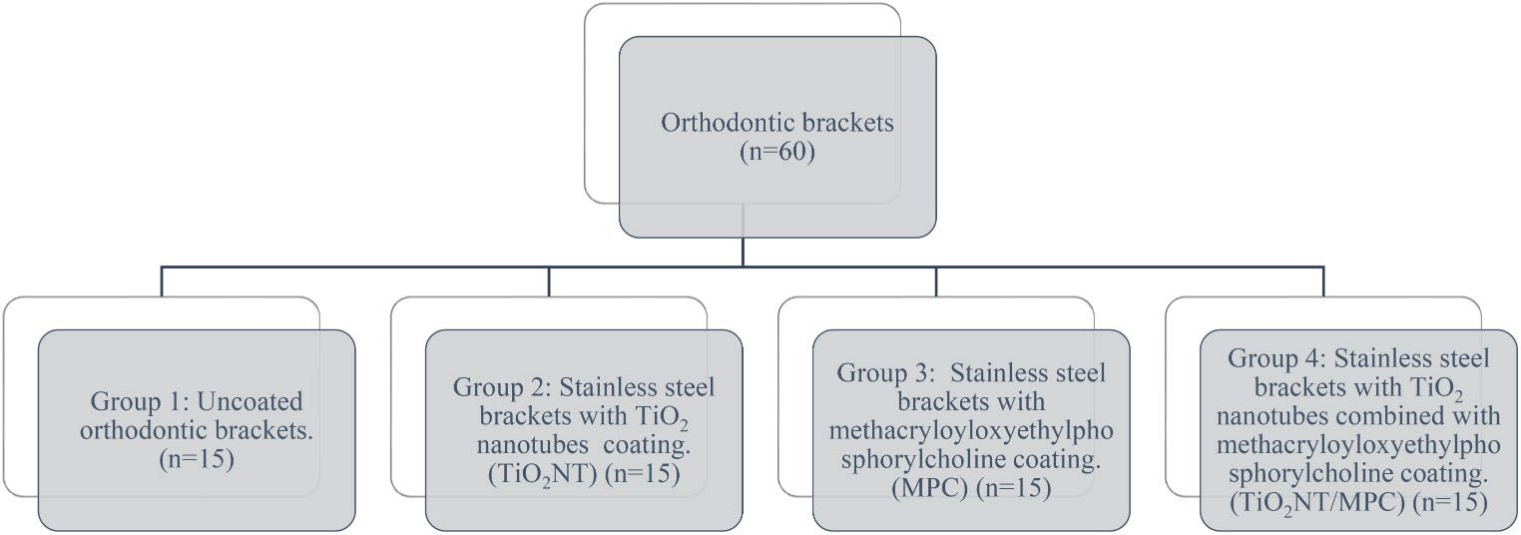
Sample size distribution

This was an in vitro study performed to assess the surface roughness, surface topography, total bacterial adhesion, colony forming units and detection of biofilm of coated and uncoated orthodontic brackets. The study sample comprised 60 stainless steel upper premolar brackets (**Desires** Ozone orthodontic bracket system, with slot dimensions 0.22” x 0.28”, M/D width of 3 mm, 80 gauge mesh, Low Profile, MBT prescription with a torque of -7 degrees and angulation of 0 degrees). Sample was distributed as in the figure. All were autoclaved, 36 were used for studying the surface characteristics, and 24 were used for studying the antibacterial properties of the coated and noncoated orthodontic brackets (Fig. 2).

**Fig. 3:**
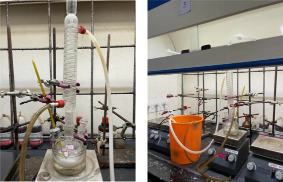
Preparation of TiO_2_ nanotubes in the lab

Uncoated brackets were used as control and for coated orthodontic brackets, 2-MPC (Sigma Aldrich) was procured, and TiO_2_NT was freshly prepared in the lab. For the preparation of TiO_2_NT, a round bottom flask was taken into which 50 ml of 10 M NaOH solution was poured and connected to a condenser along with a chilling setup. The temperature of the solution was raised to 120 °C, and 2 g of TiO2 anatase powder was added in one portion. With continuous stirring, the solution was refluxed at 120 °C for 48 h. The temperature of the mixture was reduced to 30 °C. The mixture was poured into 500 ml of deionized water. Eventually, at the bottom of the beaker, the white titanium dioxide nanotubes settled down. The water layer was drained out and washed 2 more times. To this mixture, 150 ml of 0.1 M HCl solution was added and stirred at 60 °C for 12 h. Until a neutral pH was attained, the solution was cleaned again with deionized water. The titanium dioxide nanotubes were centrifuged and calcinated at 450 °C for 4 h [26, 27] (Fig. 3).

Milli-Q water (20 mL) was placed in a Kjeldahl flask. The pH was set to 8.8 using tris base. 3 beakers were taken and to each beaker, the Milli-Q water was added, followed by the addition of 4 mg polydopamine, which served as a binder [18]. 

To the first beaker, 5 mg TiO2 nanotubes were added to the solution, the second beaker, 3 g 2-MPC was added to the solution and to the third beaker, 2.5 mg TiO2 nanotubes and 1.5 g 2-MPC was added to the solution.

**Fig. 4:**
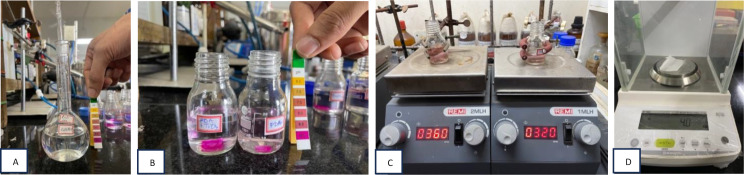
(A) 20mL Milli - Q water was taken in a kjeldahl flask. (B) pH was set to 8.8 using tris base. (C) The calculated amount of polydopamine and the antibacterial material was then weighed using a weighing scale. (D) Magnetic bead was placed into the solution with all the contents and later kept for solution stirring at around 300 rpm for 8–12 h at room temperature

A magnetic bead was placed into each beaker and later stirred at 300 rpm for 8–12 h at room temperature. Samples were recovered from the solution and placed inside the vacuum chamber. They were later transferred to labelled plastic covers until needed (Fig. 4).

### Evaluation of surface characteristics

After the coating of the orthodontic brackets, the surface topography was evaluated using scanning electron microscopy and surface roughness using atomic force microscopy.

Scanning electron microscopy with energy dispersive X-ray spectroscopy (EVO MA18 with Oxford EDS (X-act)) was used to examine the microstructure of the coated orthodontic brackets at an operating voltage of 10 kV with 2500x magnification.

To determine the surface roughness, 9 samples from each of the 36 orthodontic brackets were subjected to quantitative evaluation using atomic force microscopy (Flex-Axiom AFM, M/s Nanosurf, Switzerland). The root mean square (RMS) was measured, and an average was obtained. The images obtained depict the surface roughness at 103µm^2^.

### Evaluation of anti-bacterial characteristics

To evaluate the antibacterial characteristics, 24 orthodontic brackets of premolar teeth of the upper arch were divided into 4 groups with 6 brackets in each group. Twenty-four premolars of the upper arch extracted for orthodontic purposes were set aside and placed in distilled water (room temperature). Teeth with no carious lesions or enamel surface defects were selected. The clinical crown of each tooth was cut using diamond cutting disc, and the ends were blocked using a flowable composite (3 M Espe Filtek Supreme Flowable Restorative), phosphoric acid 37% was used to etch the facial surface of each premolar for 15 s, washed with distilled water and dried followed by application of light cure adhesive primer (3 M Transbond XT). An MBT bracket positioning gauge was used to ensure that all the brackets were placed at the desired spot on the teeth. A single observer placed the standard edgewise stainless-steel metal brackets to the centre of the facial surface of each premolar with self-cured composite (3 M Transbond XT) and excessive adhesive around the brackets were carefully removed with an explorer followed by curing of all the brackets. Then, the brackets and the teeth were sterilized.

The organism used was *Streptococcus mutans.* The source of *Streptococcus mutans* MTCC 497 was Microbial Type Culture Collection and Gene Bank (MTCC) at the Institute of Microbial Technology (IMTECH), Chandigarh, India. The organism was cultured in Brain Heart Infusion Broth for 4–6 h, and the turbidity was adjusted to 0.5 MacFarland standard to obtain a concentration of 1.5 × 10^8^ CFU/ml. The samples (tooth with the bracket belonging to each group) were placed in the wells of the tissue culture plates. Then, 2 mL of the bacterial suspension was taken and added to each sample in such a manner to completely cover it with bacterial suspension. The samples were placed inside the CO_2_ incubator at 37 °C for 24 h of incubation [28]. 

Total bacterial adhesion was measured using the optical density (OD) method. The microbial count based on spectrophotometer was done in a calibrated machine which had both positive and negative control. To remove the planktonic bacteria, the coated brackets were cleaned two times with 0.1 M phosphate buffered saline, pH 7, 20 µl of the suspension was inoculated into the microwells of the microtiter plate, which were aseptically and individually placed into a 96-well microplate. A quick read was obtained at 450 nm using a Biotek spectrophotometer [28]. Bacterial viability was assessed by counting colony forming units (CFU). After the samples were incubated, the brackets were once again cleaned with phosphate buffered saline (PBS) to eliminate the planktonic cells. Subsequently, the samples were transferred aseptically to an aseptic microcentrifuge tube consisting of 1 mL phosphate buffered saline. Vortexing was carried out at 3,300 RPM for 30s. The adhered cells were released, forming a suspension.

Logarithmic (10-fold dilutions) of the suspension was made to obtain 1 in 10, 1 in 100 and 1 in 1000 dilutions, and 10 µl of each of these dilutions was plated onto Brain Heart Infusion agar and 5% Sheep Blood agar. The plates were incubated again for 48 h at 37 °C in a 1% CO_2_ incubator. The CFU value in the brackets was calculated as the number of CFUs counted X dilution factor X 10. For inter-examiner reliability, the colony count was done by two trained observers who practiced individually in 5 independent samples [28]. 

The brackets were separated from the teeth and subjected to SEM to detect the presence of biofilm. Scanning electron microscopy with magnification set at 1000 x was used to evaluate the formation of biofilm and adhesion to orthodontics brackets. Brackets were coated with 5 nm gold (gold sputtering) to identify the bacteria.

### Statistical analysis

The data were organized and tabulated using the Microsoft Excel spreadsheet. Statistical analysis was conducted using SPSS version 20.0.2. For intragroup comparisons, Tukey’s post hoc test and one-way ANOVA were used. For intergroup comparisons, the Pearson correlation test was used. For each measurement in each group, the standard deviation, mean and mean standard error values were calculated. A p value < 0.001 was considered statistically significant.

## Results

**Fig. 5:**
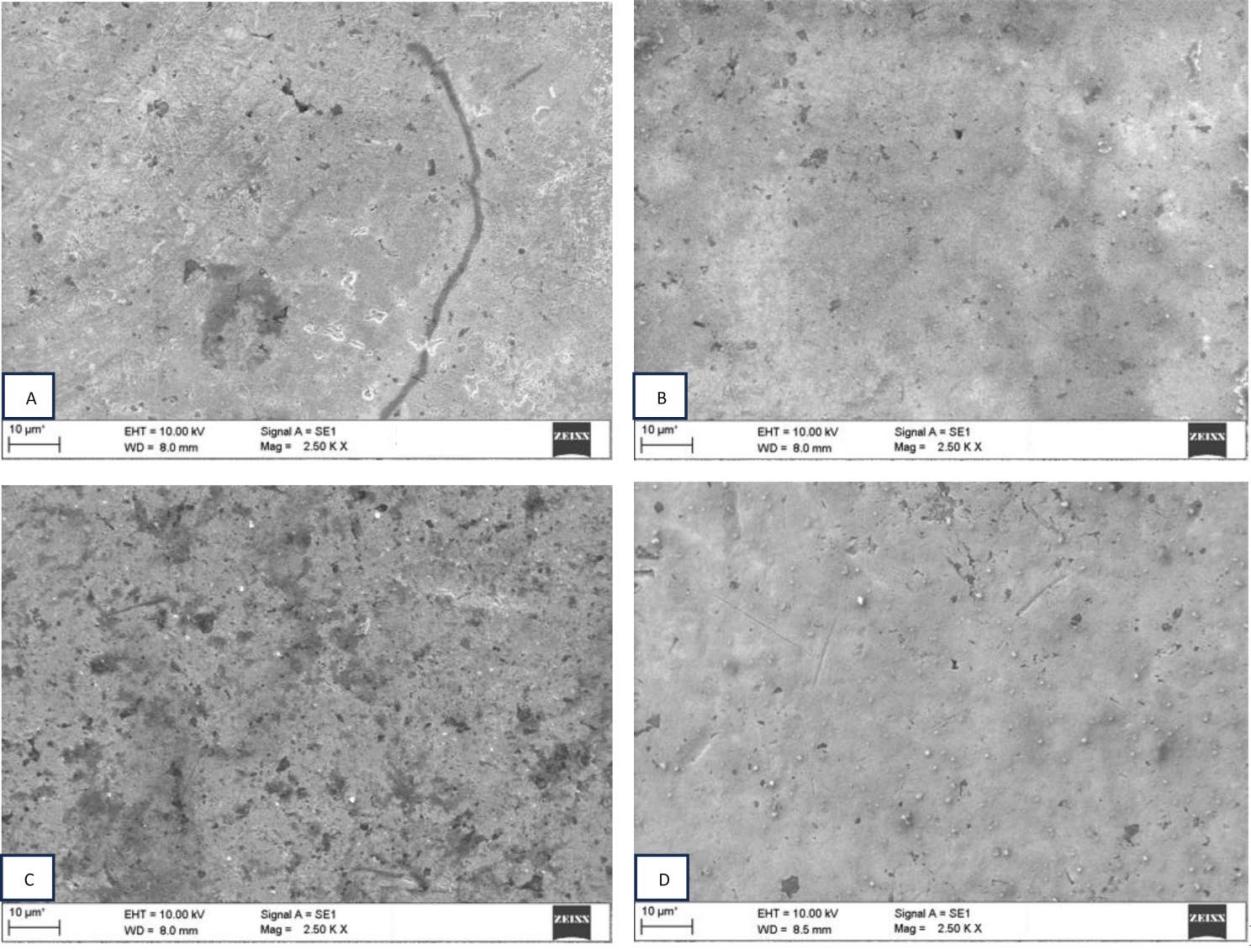
SEM images: (A) Uncoated orthodontic bracket (B) MPC coated orthodontic bracket (C) TiO_2_NT coated orthodontic bracket (D) MPC/TiO_2_ NT coated orthodontic brackets

The presence of coatings was confirmed using scanning electron microscopy (Fig. 5).

One-way ANOVA and posthoc tukey test for intra group comparisons revealed a statistically significant difference with a p value < 0.001.

Pearson correlation was performed to understand the correlation between surface roughness and antibacterial properties as given in the table. All the values depicted a positive correlation, however, not statistically significant.

Biofilm adhesion using SEM:

## Discussion

Plaques due to bacterial accumulation around the orthodontic appliances can induce the pathological process of white spot lesions. This has recently become the subject of investigation and a cause of concern. Potential bacterial retention sites in the mouth add to the growth of the biofilm, which might result in dental caries. Extensive research has contributed to the development of numerous products that patients can use to prevent or terminate demineralization.

Several authors have reported that orthodontic patients have a greater tendency to develop white spot lesions than the general population. According to Chapman 2010, clinically, white spot lesions might develop rapidly, with a significant appearance in the 4th week after initiating orthodontic treatment as a result of poor oral hygiene [29]. While white spot lesions can appear within 1 month on the areas of enamel surrounding the brackets, regular caries normally develop after at least 6 months of treatment. White spot lesions commonly emerge in the gingival area of the labial surfaces of the teeth around the brackets [30]. Tufekci et al. have stated a dramatic rise in the number of white spot lesions during the initial 6 months of treatment, with a gradual increase up to 12 months. Therefore, it is necessary to monitor patients during the 1st month of treatment to ensure that good oral hygiene is maintained. This study was conducted to address this issue and to obtain a positive outcome of minimizing white spot lesions posttreatment.

Several different fields have shown interest in bioinspired materials. Among these materials, the biomaterials used in this study are methacryloyloxyethylphosphorylcholine (MPC) and TiO_2_ nanotubes (TiO_2_NT). In this study, the relationship between the surface roughness and the antibacterial properties of coated and uncoated stainless steel orthodontic brackets were assessed. We believe this is the first study to evaluate the effect of TiO_2_ NT, MPC and TiO_2_ NT/MPC-coated stainless steel orthodontic brackets. The results disproved the null hypothesis, showing that coated orthodontic brackets have reduced surface roughness and improved antibacterial properties.

**Fig. 6:**
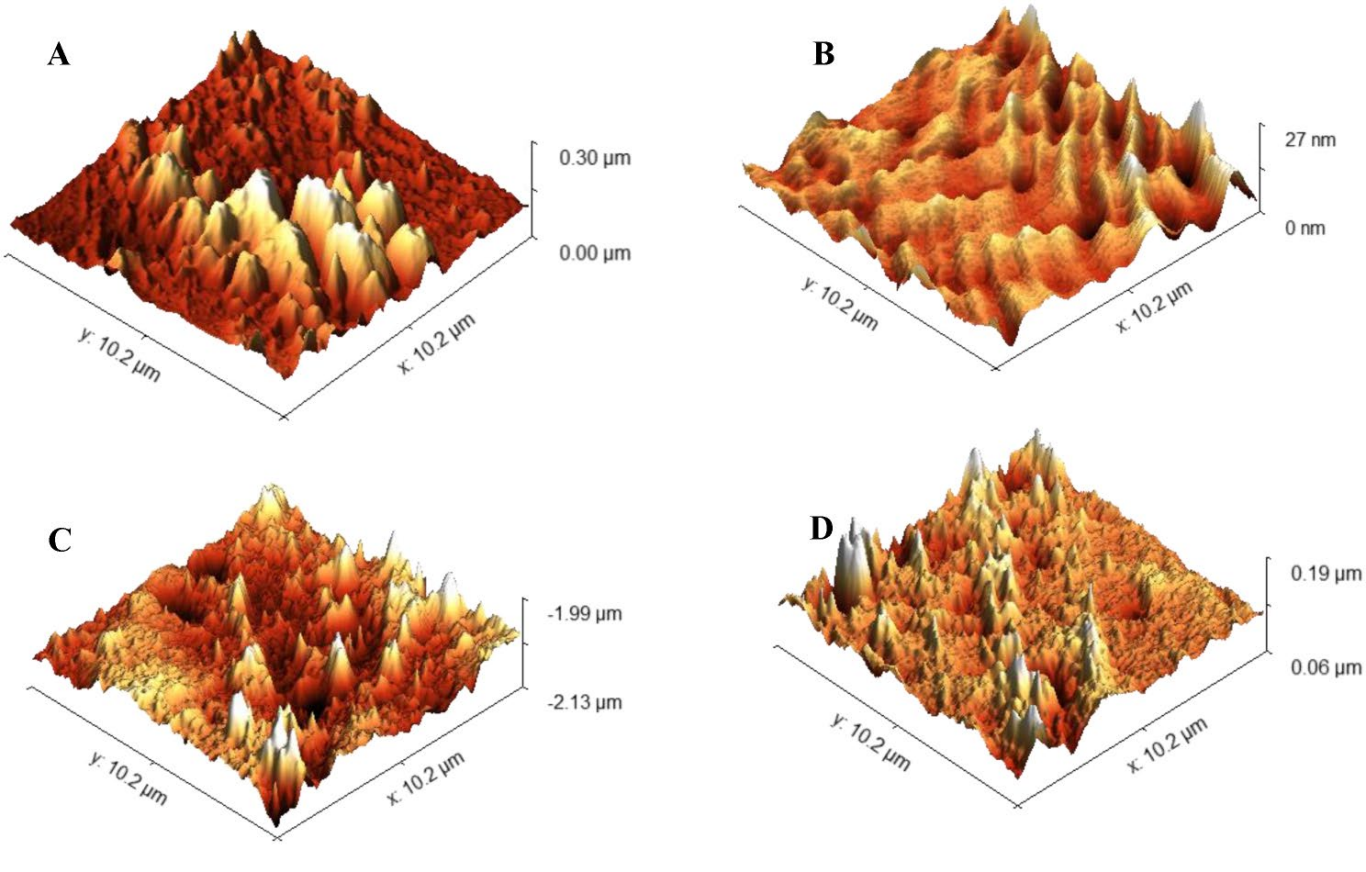
Atomic force microscopy images depicting surface roughness at 103µm^2^ : (a) Uncoated orthodontic brackets (b) TiO2 NT coated orthodontic brackets (c) MPC coated orthodontic brackets (d) TiO2 NT/MPC coated orthodontic brackets

As per the findings of the study, TiO_2_NT exhibited a surface roughness of 20–30 nm and decreased bacterial adhesion (Fig. 6). Past experiments have concluded that the antibacterial property of TiO_2_NT is determined by changes in the dimension parameters. An important dimension parameter is diameter. The hydrophilicity and surface roughness increase as the diameter increases. Peng et al. reported that TiO_2_NT 80 nm in diameter demonstrated improved antibacterial ability compared with TiO_2_NT 30 nm in diameter [31]. Simi and Rajendran concluded that TiO_2_NT with different diameters may be produced by monitoring the contents of the electrolyte. They reported that increased hydrophilicity was attributed to increasing TiO_2_NT diameters, thus refining the antibacterial property. Contradictory findings have been reported in several studies [32]. Radtke et al. suggested that TiO_2_NT formed at 5 V had smaller diameters and demonstrated better antibacterial effects than TiO_2_NT of other diameters [33]. Lewandowska et al. reported that TiO_2_NT synthesized at 4 V had diameters in the range of 20–30 nm, which yielded excellent antibacterial activity against different strains of *Staphylococcus aureus* compared to TiO_2_NT with greater diameters. The inability to accurately regulate factors during the experiments accounts for these differences. The morphology of TiO_2_NT is influenced by dimensions other than diameter and wall thickness, including length, gaps between walls, and crystal shapes. TiO_2_NT fabrication methods have limitations; therefore, precise control of the other variables to understand the effect of one parameter on the antimicrobial ability of TiO_2_NT is practically impossible. In our study, the synthesized TiO_2_NT nanotubes had widths in the range of 40–45 nm and lengths in the range of 220–250 nm, which could explain their excellent antibacterial properties.

**Fig. 7:**
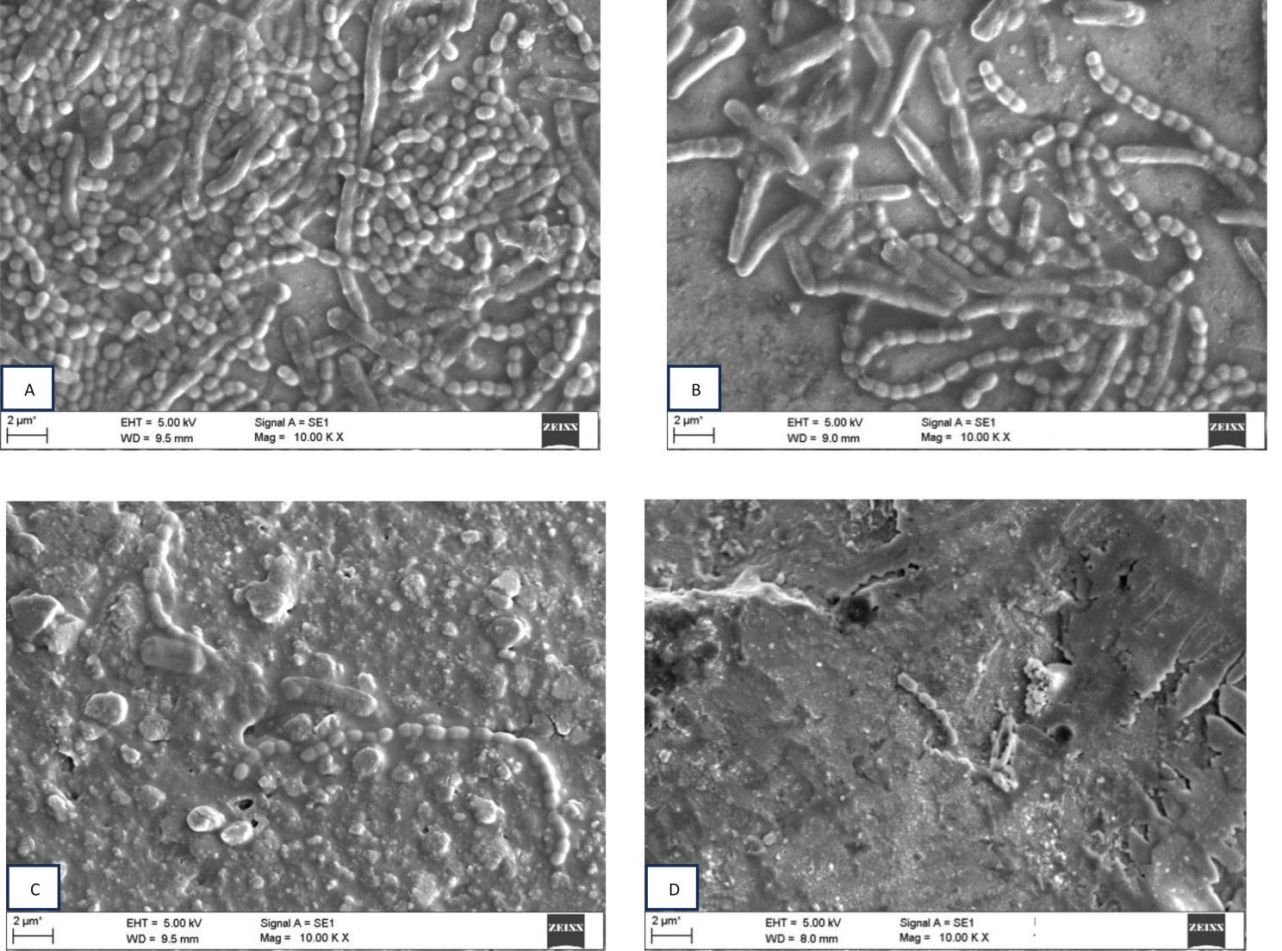
SEM images bacterial biofilm: (A) uncoated orthodontic brackets (B) MPC coated orthodontic brackets (C) TiO_2_ NT coated orthodontic brackets (D) TiO_2_ NT/MPC coated orthodontic brackets

Many studies have concluded that bacterial adhesion is mostly influenced by surface roughness [34] (Fig. 7). Findings from this study showed increased retention of bacterial plaque when the surface roughness values were equal to or greater than 0.2 μm [35]. In this study, MPC, TiO_2_NT and TiO_2_NT/MPC-coated brackets all had values below 0.2 μm, thus substantiating the antibacterial evidence obtained for all the coated orthodontic brackets (Tables 1, 2).

**Table 1:** Intra group comparisons using one-way ANOVA and posthoc tukey test

Parameters	Uncoated	MPC	TiO_2_ NT	TiO_2_ NT/MPC	P value
OD Values at 450 nm	0.08 ± 0.017	0.022 ± 0.003	0.021 ± 0.003	0.016 ± 0.004	< 0.001
Colony count (x10^8^ CFU/mL)	2.033 ± 0.446	1.017 ± 0.519	0.817 ± 0.371	0.483 ± 0.264	< 0.001
Surface roughness at 103 μm² (nm)	41.637 ± 2.437	16.969 ± 1.1	2.564 ± 0.494	12.79 ± 1.139	< 0.001

**Table 2:** Inter group comparisons using Pearson correlation for corelating the colony count 10^8^ & surface roughness at 103 μm²

SNO	BRACKETS	N	Correlation(r)	P VALUE
1	Uncoated	6	0.326	0.528
2	MPC	6	0.568	0.239
3	TiO_2_ NT	6	0.537	0.272
4	TiO_2_NT/MPC	6	0.748	0.087

There are some limitations to this study. There are several procedures to form the TiO_2_NT, and different sizes of TiO_2_NT can be obtained, however, in this study, only TiO_2_NT in the range of 40–45 nm was used, an in depth analysis maybe required to get the best sizes of TiO_2_NT to prevent specifically streptococcal adherence. Future studies with a larger sample size would strengthen the research. Investigations maybe required to consider other parameters such as leaching of the nanoparticles into the oral cavity and onto the adhesive as well as changes in the bracket surface due to repeated exposure to saliva. Since the entire bracket was coated, friction may develop between the bracket slot and the wire.

## Conclusion

Based on the observations of the present study, the following conclusions can be drawn:

- Smooth and homogenous coatings were seen on the coated orthodontic brackets, indicating decreased surface roughness.
- Coated orthodontic brackets showed decreased microbial activity when compared to coated orthodontic brackets.
- As the surface roughness of the coated orthodontic brackets decreased, the antibacterial characteristics also improved.
